# Retarded saturation of the areal capacitance using 3D-aligned MnO_2_ thin film nanostructures as a supercapacitor electrode

**DOI:** 10.1038/s41598-017-09039-x

**Published:** 2017-08-15

**Authors:** Green Kim, Ilhwan Ryu, Sanggyu Yim

**Affiliations:** Department of Chemistry, Kookmin University, Seoul, 02707 South Korea

## Abstract

The supercapacitive properties of manganese oxide (MnO_2_) thin films electrodeposited on three-dimensionally (3D) aligned inverse-opal nickel nanostructures are investigated. Compared to conventional planar or two-dimensionally (2D) aligned nanostructures, 3D-aligned nanostructures can provide considerably increased and controllable contacts between the electrode and electrolyte. As a result, saturation of the areal capacitance with the electrode thickness and associated decrease of the specific capacitance, *C*_*sp*_, become much slower than those of the planar and 2D-aligned electrode systems. While, for planar MnO_2_ electrodes, the *C*_*sp*_ of a 60-cycle electrodeposited electrode is only the half of the 10-cycle electrodeposited one, the value of the 3D-nanostructured electrode remains unchanged under the same condition. The maximum *C*_*sp*_ value of 864 F g^−1^, and *C*_*sp*_ retention of 87.7% after 5000 cycles of galvanostatic charge-discharge are obtained. The voltammetric response is also improved significantly and the *C*_*sp*_ measured at 200 mV s^−1^ retains 71.7% of the value measured at 10 mV s^−1^. More quantitative analysis on the effect of this 3D-aligned nanostructuring is also performed using a deconvolution of the capacitive elements in the total capacitance of the electrodes.

## Introduction

**Figure 1:**
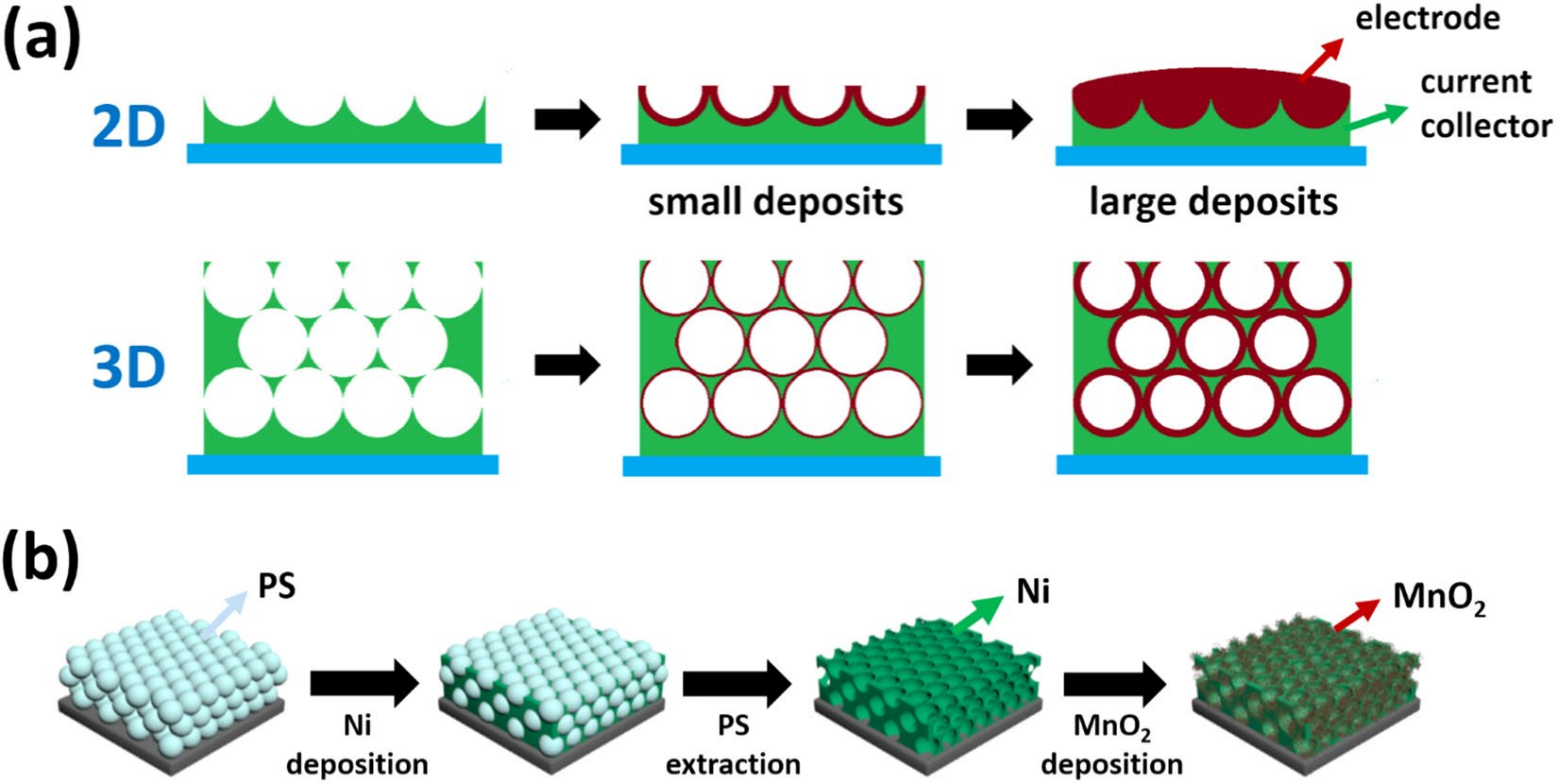
Schematic illustration of the fabrication of nanostructured MnO_2_ electrtodes. (**a**) (upper) 2D- and (lower) 3D-aligned current collector/electrode systems with small and large deposits of electrode materials. For the 3D-aligned architecture, the increase in thickness of the electrode layer can be retarded by piling up the current collector nanostructures. (**b**) Schematic illustration representing the fabrication of 3D-aligned MnO_2_ thin film nanostructures in this work.

Pseudocapacitors, or supercapacitors that use transition metal oxides and conducting polymers as electrode materials, are attracting growing attention due to their superior specific energy and capacitance compared to the current electrochemical double layer capacitors (EDLCs)^1–3^. Among the various transition metal oxides, manganese oxide (MnO_2_) is considered one of the most promising electrode materials due to its high theoretical capacitance, environmentally friendly characteristics and low price^4–6^. However, the specific capacitance, *C*_*sp*_, values of the MnO_2_ electrode reported are far below the theoretical value of 1370 F g^−1^ ^4^ because of its poor electrical conductivity (10^−5^–10^-6^ S cm^−1^)^7^. In this context, the fabrication of various types of MnO_2_ nanostructures such as nanoparticles^8^, nanowires^9, 10^, and nanotubes^11^ was investigated aiming for the easier charge-transport between the electrode and electrolyte. However, the increases in the capacitance of the electrodes were limited, and the *C*_*sp*_ values reported were only in the range of 220 – 355 F g^−1^. Instead of those unaligned nanostructures, two-dimensionally (2D) arrayed MnO_2_ nanostructures were also proposed^12, 13^ since it is generally accepted that aligned electrode architectures are superior to unaligned ones by providing highly-organized charge pathways and tunable interspaces between the architecture units^14^. For example, the deposition of MnO_2_ on 2D-arrayed nickel (Ni) nanocones exhibited a significant improvement in *C*_*sp*_ values to 632 F g^−1^ and a cycle performance of 95.3% retention after 20,000 cycles^12^. More recently, the *C*_*sp*_ value of MnO_2_ thin film nanostructures fabricated by electrodepositing MnO_2_ on 2D-arrayed polystyrene (PS) nanosphere templates, followed by the removal of the PS spheres, was reported to be 714 F g^−1^ ^13^. Although the specific capacitance of these 2D-aligned nanostructures has increased to some extent, the advantages of the nanostructures were only realized for extremely small deposits of electrode materials as illustrated in Fig. 1a. This is because as the deposition proceeds, the 2D-aligned nanostructure is gradually filled and covered with the electrode material, and finally buried under a planar electrode layer. Consequently, the areal capacitance of the electrode is easily saturated and the *C*_*sp*_ value decreases rapidly as the MnO_2_ film thickness increases. This indicates that conventional two-dimensional nanostructures are not suitable for obtaining sufficient specific energy necessary for practical use because the nanostructuring effect can be exerted only when the extremely small amount of electrode materials is used.

To solve these problems, we propose a new technique to fabricate three-dimensionally (3D)-aligned MnO_2_ nanostructure electrodes by electrodepositing MnO_2_ thin films onto 3D-arrayed Ni inverse-opal nanostructures. The 3D-arrayed Ni nanostructure was easily fabricated by well-known colloidal nanosphere lithography and its height could be controlled by the number of nanosphere stacks, implying that the electrode with a desired amount of electrode material can be fabricated while maintaining the effect of nanostructure. It was observed that this 3D-aligned architecture retarded the saturation of the areal capacitance and the associated decrease in the specific capacitance of the electrode considerably. The voltammetric response and cycle life of the electrode were also much improved.

## Results

**Figure 2:**
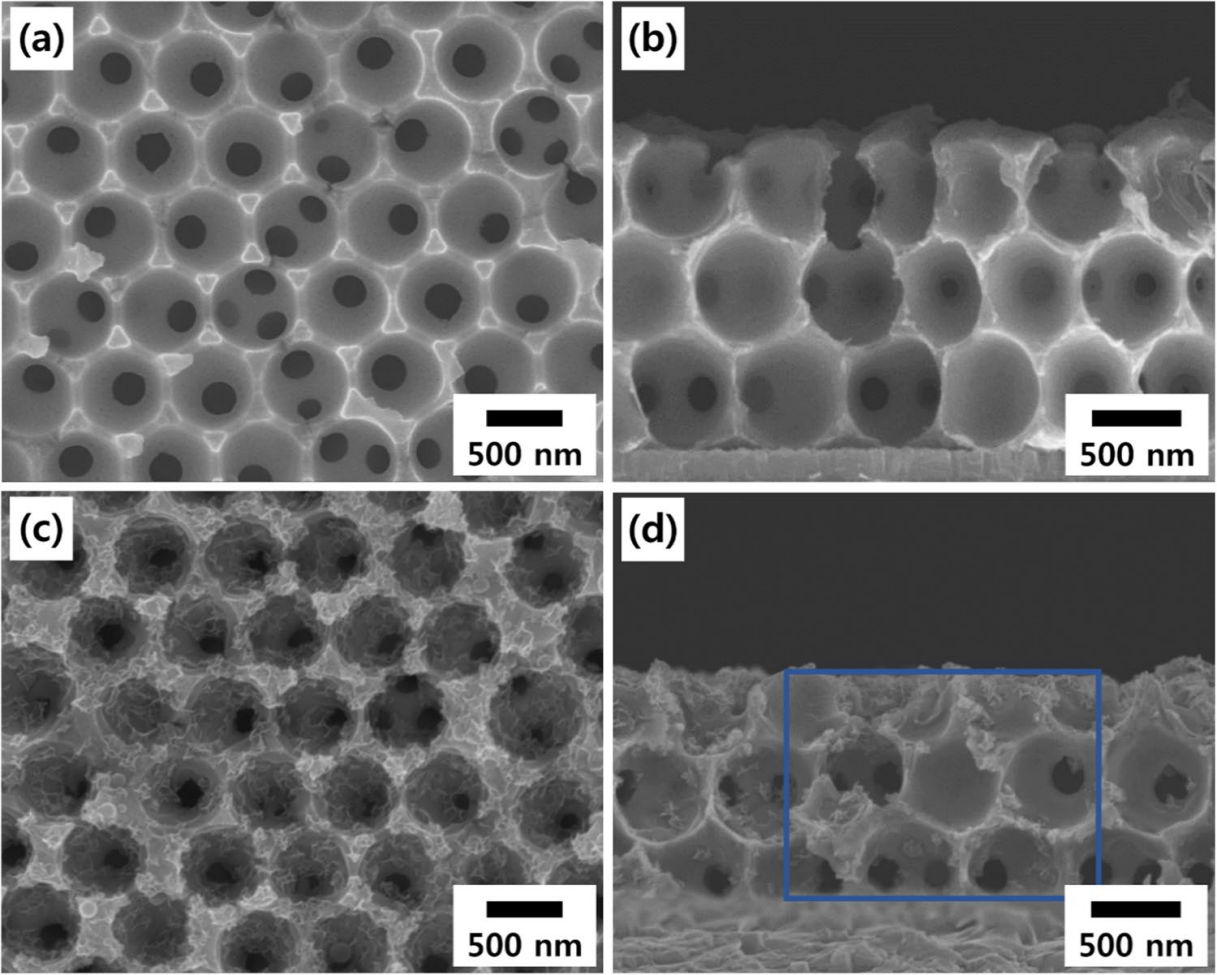
Morphology characterization of the current collector and electrode nanostructures. (**a**) Surface and (**b**) cross-sectional FE-SEM images of 3Lyr inverse-opal Ni nanostructures, and (**c**) surface and (**d**) cross-sectional FE-SEM images after 10 cycle electrodeposition of MnO_2_ on the Ni nanostructures. Elemental mapping images of the marked area in (**d**) are represented in the Supplementary Information (Figure S4).

**Figure 3:**
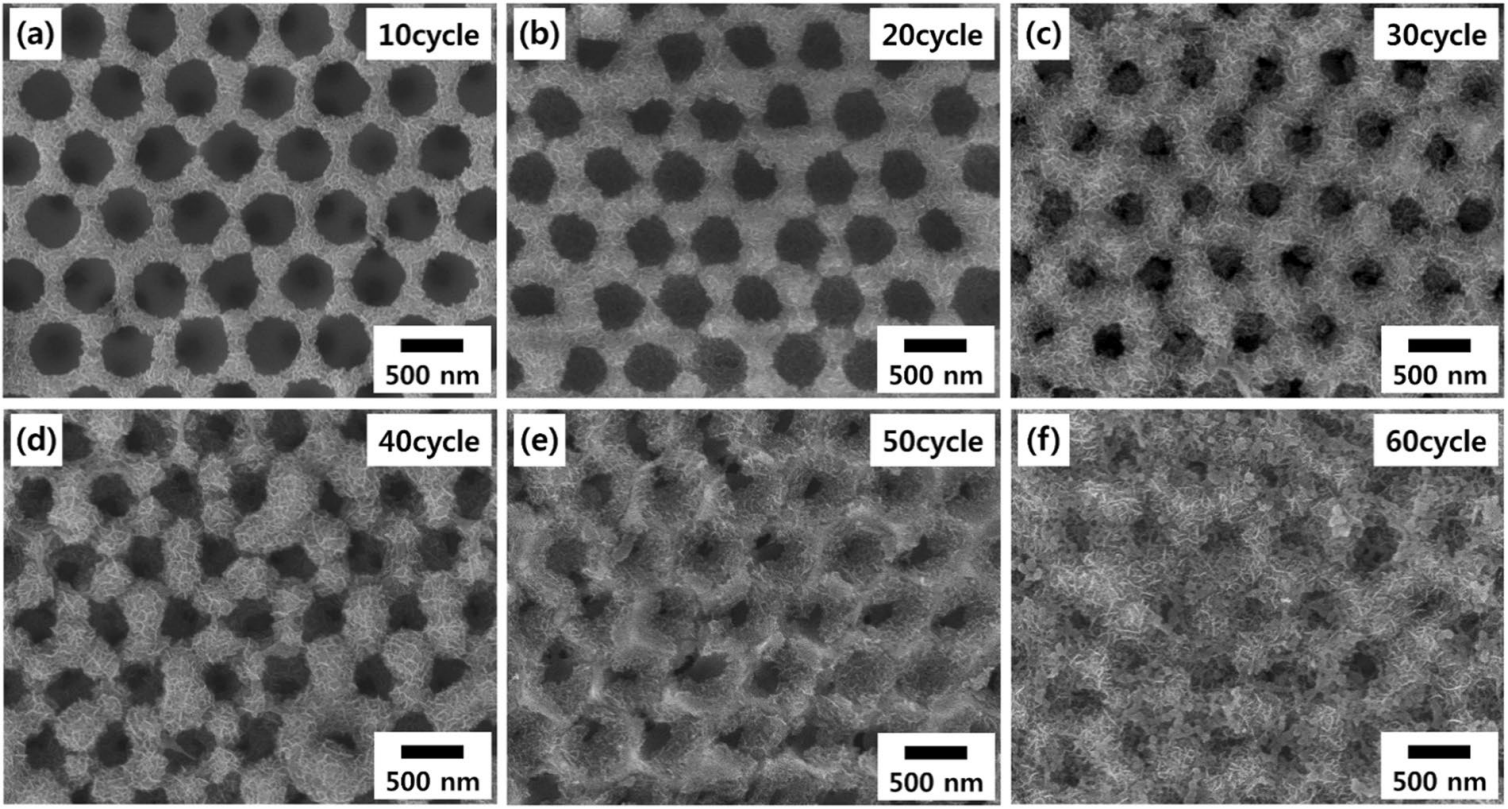
Surface morphology of the nanostructured MnO_2_ electrodes. Surface FE-SEM images of MnO_2_ thin films electrodeposited on a 5Lyr inverse-opal Ni nanostructure. The number of electrodeposition cycles is (**a**) 10, (**b**) 20, (**c**) 30, (**d**) 40, (**e**) 50 and (**f**) 60.

Figure 2a and b show the surface and cross-sectional field emission scanning electron microscopy (FE-SEM) images of a three layer (3Lyr)-stacked inverse-opal Ni nanostructure with a periodic dimension of 580 nm. The 3D nanostructure interconnecting spherical voids was formed by cathodic deposition of Ni onto 3D-arrayed PS nanospheres, followed by the removal of the PS nanospheres. This Ni nanostructure was then used not only as a working electrode during the electrodeposition of MnO_2_ thin films on it but also as a current collector for the fabricated supercapacitors in this study. The formation of the MnO_2_ thin films on the inverse-opal Ni nanostructure was performed by cyclic voltammetric deposition in a MnSO_4_·H_2_O aqueous solution. The surface and cross-sectional FE-SEM images of 15 cycle-electrodeposited MnO_2_ electrodes are shown in Fig. 2c and d, respectively. The repeated voltammetric cycles led to a continuous deposition of MnO_2_ on the Ni nanostructure. The morphological evolution of the surface of a five layer (5Lyr) nanostructure at various numbers of the electrodeposition cycles is shown in Fig. 3. The nanowires, each with a width of a few nanometers, characteristic of electrodeposited MnO_2_^9, 13, 16^, were clearly observed in all cases. The thickness of the MnO_2_ layer gradually increased and after 60 cycles of electrodeposition, the MnO_2_ nanowires almost covered the entire void of the film, as shown in Fig. 3f. For the 3Lyr electrode, a similar entire coverage was obtained after 30 cycles (Figure S2), indicating that MnO_2_ was electrodeposited constantly and uniformly on the Ni nanostructures. The cross-sectional FE-SEM images (Fig. 2d and Figure S3) and elemental mapping results for Mn atoms (Figure S4) also showed the infiltration of the MnO_2_ in the interstitial spaces of the Ni nanostructure from the bottom to the top. This uniform deposition of the MnO_2_ implies that the amount and areal capacitance of the MnO_2_ electrode can be extended continuously by increasing the number of stacking layers of the 2D-arrayed PS template while preserving the electrode thickness and advantages of the nanostructure. It is very important for the practical use of nanostructured pseudocapacitor electrodes that a new current collector nanostructure is continuously provided and MnO_2_ is uniformly deposited thereon. This is because, for areal capacitance easily saturates and hence the specific capacitance rapidly decreases as the electrode thickness increases, making it difficult to obtain desired specific energy. To estimate the effect of the 3D nanostructures, a planar MnO_2_ thin film electrode was also fabricated and investigated. The surface morphologies of the planar Ni film and MnO_2_ layer deposited on it are shown in Figure S5a and b, respectively. The weight of the electrodeposited MnO_2_ increased almost linearly and equally on both the planar and nanostructured Ni current collectors as the deposition cycle proceeded, reaching approximately 45 μg cm^−2^ after 60 cycles of deposition (Figure S6).

**Figure 4:**
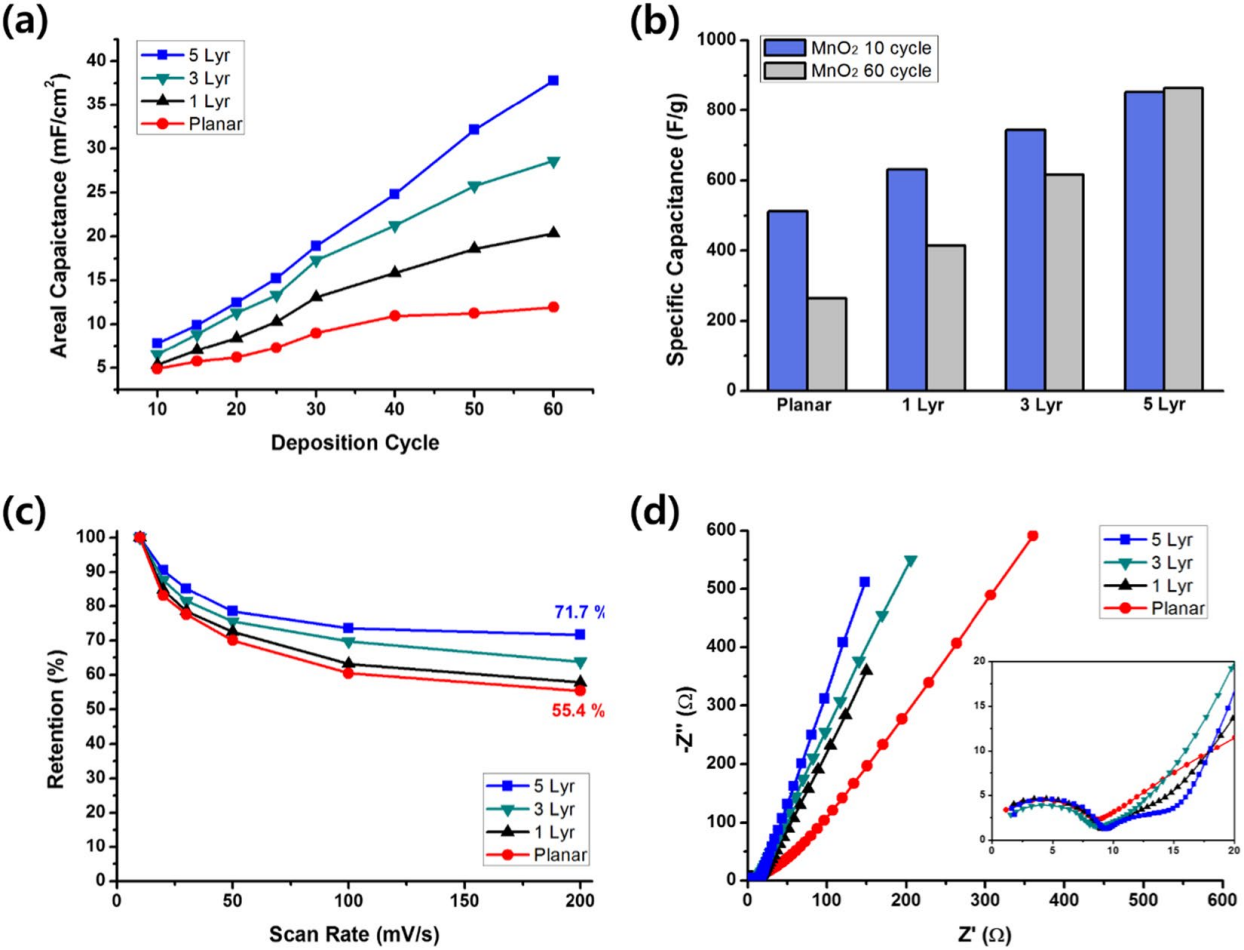
Electrochemical characterization of MnO_2_ electrodes at various numbers of electrodepoistion cycles and scan rates. (**a**) Plots of the areal capacitance as a function of the number of MnO_2_ electrodeposition cycles and (**b**) bar graphs of the specific capacitances after 10 and 60 electrodeposition cycles. The scan rate was 100 mV s^−1^ for these measurements. (**c**) Plots of specific capacitance retentions as a function of the scan rate and (**d**) Nyquist plots for the planar and nanostructured electrodes. The number of MnO_2_ electrodeposition cycles was 60 for these measurements.

The electrochemical performance of the half-cell supercapacitors based on both planar and 3D-nanostructured MnO_2_ electrodes was estimated by cyclic voltammetry (CV) measurements performed in a 1.0 M aqueous Na_2_SO_4_ solution within a potential range from 0.0 V to 0.8 V. Figures. S7a and S7b show the CV contours measured at a scan rate of 100 mV s^−1^ for the planar and 5Lyr nanostructured electrodes, respectively, fabricated with various cycles of MnO_2_ electrodeposition. The almost symmetric CV curves for both cases indicate the reversibility of the redox transition of MnO_2_. Figure 4a represents the plots of the areal capacitances as a function of the number of MnO_2_ electrodeposition cycles. The areal capacitance of the 3D-nanostructured electrodes exceeded the value of the planar electrode at all deposition cycles, and the difference was larger along with the number of layers of the 3D nanostructures. More interestingly, while the areal capacitance of the MnO_2_ electrode on the planar Ni film was saturated after approximately 40 deposition cycles, the saturation of the areal capacitance for the 3D-nanostructured electrodes took a much greater number of cycles. In the case of the 5Lyr electrode, the increase was almost proportional to the number of electrodeposition cycles and saturation has still not been achieved after 60 cycles (blue squares in Fig. 4a). This is probably because that the bottom part of the MnO_2_ layer on the planar Ni film became less electrochemically accessible to the electrolyte as the deposition proceeded. In contrast, for the MnO_2_ electrodes on the 3D Ni nanostructures, the thickening of the MnO_2_ layer can be retained by piling up the stacks of the Ni nanostructures, thus providing new surface area for MnO_2_ deposition. The specific capacitance, *C*_*sp*_, was calculated by the following equation,

1 $${C}_{sp}=\frac{I}{m(dV/dt)}$$

where *I* (A) is the average current, *m* (g) is the mass of deposited MnO_2_, and *dV/dt* (mV s^−1^) is the scan rate^17^. In the case of the electrode on the planar Ni film, the *C*_*sp*_ value gradually decreased as the number of deposition cycles increased. The specific capacitance of the 60-cycle deposited electrode was 264 F g^−1^, and is only 51.6% of the *C*_*sp*_ value after 10-cycles (512 F g^−1^) as shown in Fig. 4b. In contrast, for the nanostructured electrodes, the decrease in the *C*_*sp*_ value at large MnO_2_ deposition cycles was reduced considerably. For MnO_2_ electrodes on the 1 Lyr and 3 Lyr Ni nanostructures, the *C*_*sp*_ values of the 60-cycle deposited electrodes were 65.7% and 82.8% of the *C*_*sp*_ values of the 10-cycle deposited electrodes, respectively. In the case of the 5Lyr electrode, the *C*_*sp*_ value of the 60-cycle deposited electrode, 864 F g^−1^, was even slightly larger than that of the 10-cycle deposited electrode, 852 F g^−1^. This proportional increase in areal capacitance and retarded decrease in specific capacitance can be attributed to the benefits of 3D-aligned nanostructures, *i.e*. more effective ion and charge transport along with a considerably increased surface area of the electrodes.

Another benefit of the nanostructured electrodes is the rapid voltammetric response. Figure S8 shows CV curves at various scan rates ranging from 10 mV s^−1^ to 200 mV s^−1^ for the planar and nanostructured electrodes. Figure 4c shows plots of areal capacitance retention as a function of scan rates. In the case of the planar electrode, the areal capacitance of 15.4 mF cm^−2^ at a scan rate of 10 mV s^−1^ dropped to 8.5 mF cm^−2^ at 200 mV s^−1^, and hence the retention was 55.4%. The retentions of the 3D-nanostructured electrodes gradually increased as the height of the nanostructure increased. For 1Lyr and 3Lyr electrodes, the retentions were 57.9% and 63.8%, respectively. The areal capacitance of the 5Lyr electrode was 38.2 mF cm^−2^ at a scan rate of 10 mV s^−1^, which decreased to 27.4 mF cm^−2^ at 200 mV s^−1^, and the retention was 71.7%. This enhancement in the voltammetric response of nanostructured electrodes can also be attributed to the efficient access of ions to the electrode surface and transport of charge carriers to the current collectors. Nyquist plots of planar and 3D-nanostructured electrodes obtained in the frequency range from 10^−1^ to 10^5^ Hz are shown in Fig. 4d. The Z’ and Z” values are the real and imaginary part of the impedance, respectively. The diameter of the semicircle in the high-frequency region, which is related to the charge-transfer resistance, *R*_*ct*_, did not change apparently as shown in the inset of the figure. This is probably because the charge transfer is affected mainly by the oxidation states of manganese, not by the electrode morphology^18, 19^. In contrast, the slopes of the line in the low-frequency region varied considerably. The slope is inversely related to the diffusive resistance, *R*_*d*_, and the steeper line of the nanostructured electrode reflects a smaller resistance due to the better diffusion on the 3D-aligned porous nanostructures^20–22^.

**Figure 5:**
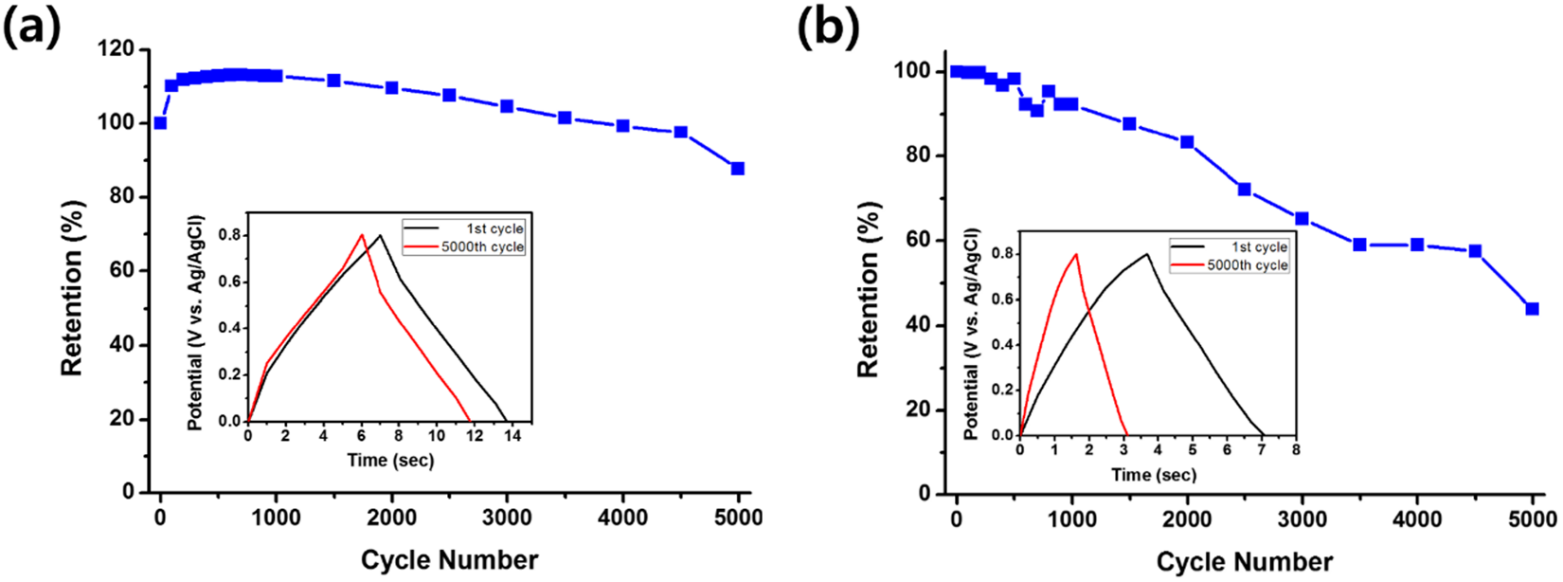
Cyclic stability of MnO_2_ electrodes. Plots of *C*_*sp*_ retentions for (**a**) 5Lyr nanostructured and (**b**) planar MnO_2_ electrodes as a function of the number of galvanostatic charge-discharge cycles. The number of MnO_2_ electrodeposition cycles was 60 for both electrodes. (Inset) Galvanostatic charge-discharge curves measured after 1 and 5000 cycles at a constant current density of 1.0 mA cm^−2^.

The long-term stability of the 3D-nanostructured MnO_2_ electrodes was examined in a 1.0 M Na_2_SO_4_ aqueous electrolyte by applying a continuous galvanostatic charge-discharge cycle at a current density of 1.0 mA cm^−2^. Fig. 5a and b represent the *C*_*sp*_ retentions as a function of galvanostatic charge-discharge cycles for the 5Lyr-nanostructured and planar MnO_2_ electrode, respectively, and the insets show the galvanostatic curves measured after 1 and 5000 charge-discharge cycles. The relatively unchanged shape of the curve and large *C*_*sp*_ retention of 87.7% for the nanostructured electrodes (Fig. 5a) indicate a superior long-term stability, which can also be attributed to the 3D-aligned nano-architecture. In contrast, the *C*_*sp*_ of the planar electrode decreased rapidly with the number of charge-discharge cycles and dropped 43.9% of the initial value after 5000 charge-discharge cycles as shown in Fig. 5b, indicating that some of the MnO_2_ deposits are detached from the Ni current collector during the charge-discharge cycles. This result implies that the 3D-aligned nano-architecture of the electrodes can also provide a solution to the detachment problem of the MnO_2_ electrodes from the current collector surface, without the need to use undesirable polymeric binders.

**Figure 6:**
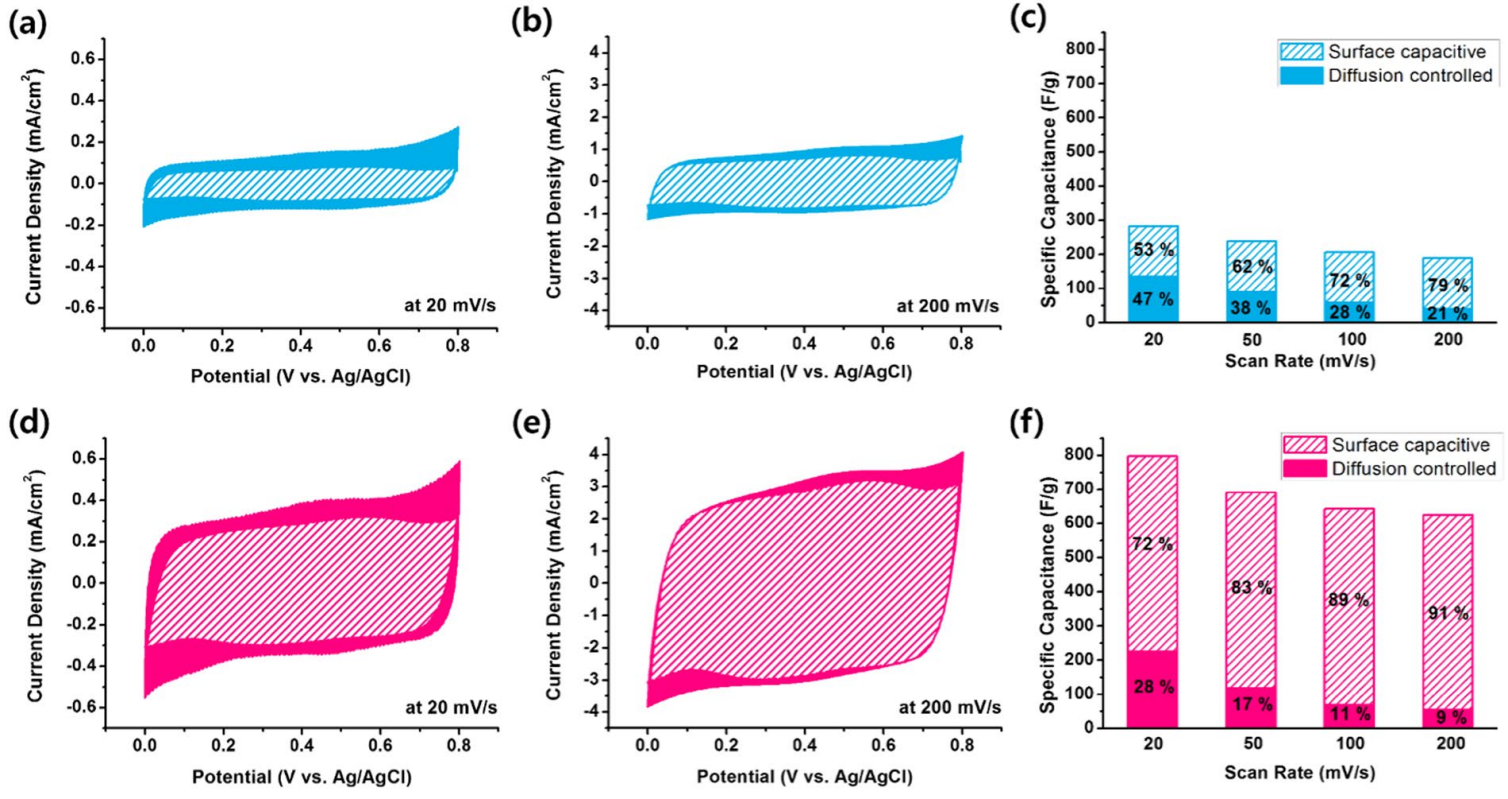
Extraction of capacitive elements. Deconvolution of the total capacitance into surface capacitive (shaded part) and insertion (solid part) elements for the planar MnO_2_ electrode at scan rates of (**a**) 20 mV s^−1^ and (**b**) 200 mV s^−1^, and for the 5Lyr nanostructured MnO_2_ electrode at scan rates of (**d**) 20 mV s^−1^ and (**e**) 200 mV s^−1^. The bar graphs represent the specific capacitance of each of the two elements for the (**c**) planar and (**f**) 5Lyr nanostructured MnO_2_ electrodes at various scan rates.

Further quantitative estimation of the nanostructuring effect on the capacitive properties of the MnO_2_ electrodes was carried out using a deconvolution method proposed by Conway^23^ and Dunn^24^. A current at a given voltage can be divided into two elements from the deconvolution, namely, a surface capacitive element (*k*_1_ *v*) and Faradaic insertion element (*k*_2_ *v* ^1*/*2^) by using the following equation;

2 $$i(V)={k}_{1}v+{k}_{2}{v}^{1/2}$$

where *i(V)* is the current, *v* is the scan rate, and *k*_1_ and *k*_*2*_ are constants. The details of the process to extract the elements are described elsewhere^25^. The *k*_*1*_ *v* plots for the planar MnO_2_ electrode at low (20 mV s^−1^) and high (200 mV s^−1^) scan rates are represented by shaded areas in Fig. 6a and b, respectively. The remaining area of the total CV graph (blue solid part) corresponds to the Faradaic insertion element. The contributions of the two elements in the total capacitance at various scan rates were calculated and are presented in Fig. 6c. The surface capacitive element (blue shaded part) denotes the sum of double-layer charging and surface Faradaic pseudocapacity. The value of 149 F g^−1^ obtained for the planar MnO_2_ electrodes was virtually independent of the scan rate^26^. In contrast, the contribution of the Faradaic insertion capacity (blue solid part) gradually decreased with the scan rate, from 134 F g^−1^ at 20 mV s^−1^ to 40 F g^−1^ at 200 mV s^−1^. This decrease is because the diffusion-controlled insertion becomes less accessible at higher scan rates, consistent with previous reports^16, 25, 26^. The contribution of the insertion element in the total capacitance of the planar MnO_2_ electrodes was 47% at a scan rate of 20 mV s^−1^ and 21% at a scan rate of 200 mV s^−1^. These values are slightly smaller than the values previously reported for nanostructured MnO_x_ electrodes, *i.e*. 60% at 5 mV s^−1^ and 25% at 100 mV s^−1^ for mesoporous MnO_2_ nanowires^16^ and 66% at 20 mV s^−1^ and 55% at 100 mV s^−1^ for Mn_3_O_4_ nanoparticles^25^. In contrast, in the case of the 5Lyr-nanostructured electrode, the contribution of the surface capacitive element (pink shaded part) was considerably larger at the entire range of scan rates, as shown in Fig. 6d–f. The surface capacitive element was 573 F cm^−2^ and was also independent of the scan rate. This value is approximately four times the value for the planar MnO_2_ electrode. However, the increases in the insertion element were not significantly greater. As a result, the contribution of the insertion element in the total capacitance of the 5Lyr nanostructured MnO_2_ electrodes was only 28% at a scan rate of 20 mV s^−1^ and 9% at a scan rate of 200 mV s^−1^. Additional deconvolution data for the electrodes at other scan rates are shown in the Supplementary Information (Figure S9). The significantly increased surface capacitive elements indicate that the 3D-aligned nanostructures are effectively well fabricated and due to the increased contact with electrolyte the surface capacitive elements became more dominant in the total capacitance.

**Figure 7:**
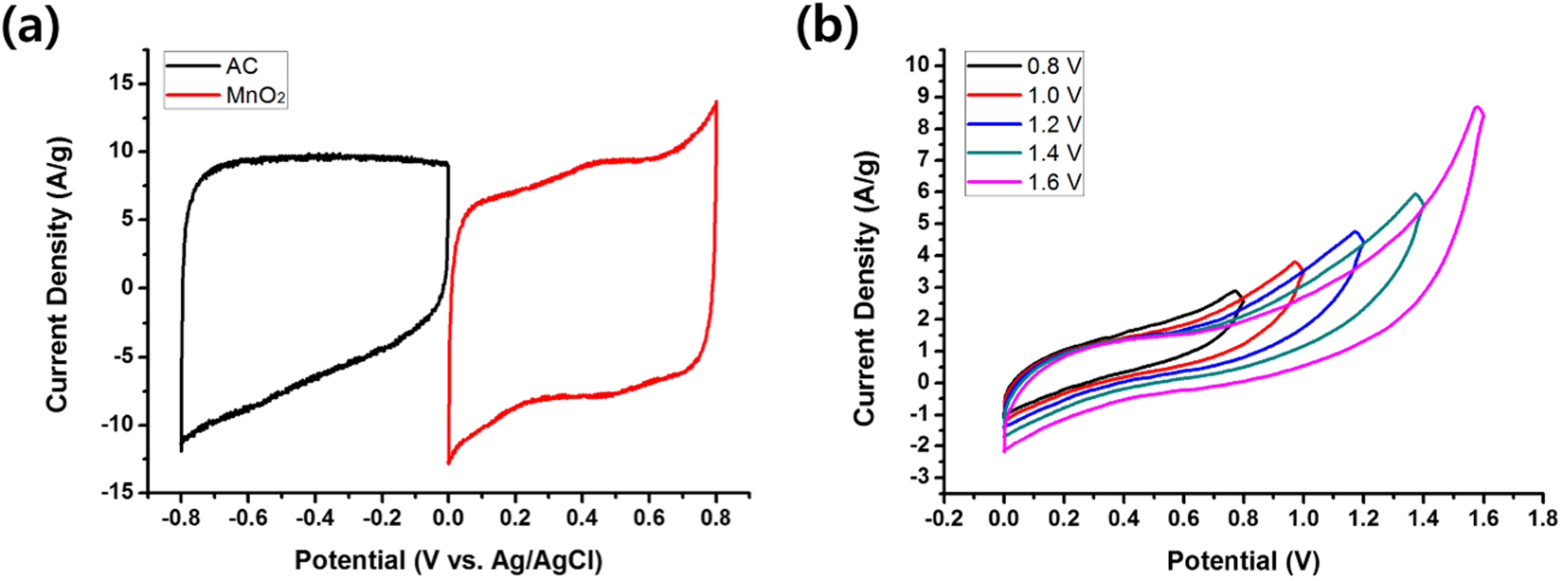
Characterization of asymmetric AC//MnO_2_ two-electrode supercapacitor. Cyclic voltammograms of (**a**) individual AC and MnO_2_ electrode measured in a three-electrode system at a scan rate of 20 mV s^−1^ and (**b**) asymmetric AC//MnO_2_ supercapacitors measured at a scan rate of 20 mV s^−1^ with various potential windows.

A two-electrode supercapacitor cell based on the 3D-aligned MnO_2_ nanostructure as a positive electrode and activated carbon (AC) as a negative electrode was also assembled since such an asymmetric supercapacitor is a promising design strategy for high voltage and energy density^27^. The mass ratio of MnO_2_ and AC was determined by *C*_*sp*_ values of individual half-cell electrode measured in a three-electrode system at a scan rate of 20 mV/s (Fig. 7a) and the following equation;

3 $$R=\frac{{m}_{+}}{{m}_{-}}=\frac{{C}_{-}{\rm{\Delta }}{V}_{-}}{{C}_{+}{\rm{\Delta }}{V}_{+}}$$

where *m* is the mass, *C* is the specific capacitance, Δ*V* is the potential window, and + and – denote the values for positive and negative electrodes, respectively. The CV curve of the AC negative electrode in the potential window of –0.8 to 0 V shows a nearly rectangular shape without noticeable redox peaks, indicating a typical characteristic of EDLC. In contrast, the CV curve of the MnO_2_ positive electrode in the potential window of 0 to 0.8 V deviates slightly from the ideal rectangular shape, indicating its characteristic of pseudo-capacitance. Figure 7b shows CV curves of the asymmetric supercapacitor device based on AC//MnO_2_ electrodes at various potential windows. The used amounts of AC and MnO_2_ were 80 μg and 44 μg, respectively. The maximum potential window of 1.6 V was determined by the results of the individual half-cell electrode. As the potential window increased, the area of the CV contours increased and the energy density of the cell reached 36.6 Wh kg^−1^ at the potential window of 1.6 V. The energy density of the asymmetric cell was calculated using the following equation.

4 $$E=\frac{1}{2}C{({\rm{\Delta }}V)}^{2}$$

As compared to the energy densities of 10.4–25.2 Wh kg^−1^ for previously reported asymmetric AC//MnO_2_ capacitors^28–31^, significantly larger energy density in this work indicates that the MnO_2_ thin film electrodeposited on the inverse opal Ni nanostructure is a promising electrode-current collector system for high energy asymmetric supercapacitors.

## Discussion

The 3D-aligned MnO_2_ electrodes for pseudocapacitors were successfully fabricated by electrodepositing MnO_2_ nanowires on an inverse-opal Ni nanostructure. The Ni nanostructure was prepared by cathodic deposition of Ni on hexagonal close-packed 3D PS nanospheres, followed by extraction of the PS. Compared to the planar and 2D-nanostructured electrodes, this 3D-aligned porous nanostructure system could continuously provide additional electrode surface by stacking up the PS template layer-by-layer. As a result, an increase in the areal capacitance was almost proportional to the thickness of the electrode and the decrease in the specific capacitance, *C*_*sp*_, was retarded significantly. While the *C*_*sp*_ values of the 60-cycle electrodeposited MnO_2_ electrodes for the planar and 2D-aligned (1Lyr) electrode were 51.6% and 65.7% of those of 10-cycle electrodeposited electrodes, respectively, that for the 3D-aligned (5Lyr) electrode was 101.4%. The voltammetric response was also improved. The *C*_*sp*_ value of the 5Lyr electrode measured at a scan rate of 200 mV s^−1^ retained 71.7% of the value measured at 10 mV s^−1^, which is significantly larger than the retention of 55.4% observed for the planar electrode at the same scan rate condition. The electrochemical impedance spectroscopy (EIS) measurements indicated that the 3D-aligned nanostructure lowered the diffusive resistance of the electrode, which contributed to an improvement of the capacitive properties. The effect of the 3D-aligned nanostructuring was also analysed by deconvoluting the capacitive elements of the electrodes. The significantly large surface capacitive elements of the 5Lyr electrode compared to those of the planar electrode indicated that the 3D-nanostructured electrodes were successfully fabricated and their contact with electrolyte was highly effective. All the results presented here demonstrate that the benefits of using nanostructures for supercapacitors, *i.e*. large contact area and effective diffusion of ions, are maximized by the 3D-aligned porous nanostructures.

## Methods

### Fabrication of 3D inverse-opal Ni nanostructures

The basic strategy to fabricate 3D-aligned MnO_2_ thin film nanostructures is illustrated in Fig. 1b. First, the template layer of 2D close-packed PS nanospheres with a diameter of 580 nm (Interfacial Dynamics Co.) was transferred onto a thoroughly cleaned fluorine-doped tin oxide (FTO)-coated glass substrate using a scooping transfer technique^15^. This transfer process was repeated layer-by-layer to stack PS nanosphere layers and attain the desired height for a 3D-nanostructured electrode. Then, an inverse-opal Ni current collector nanostructure was prepared on this 3D-arrayed PS opal template. The template was put into a NiSO_4_·6H_2_O/NiCl_2_·6H_2_O/boric acid/ethanol/H_2_O (0.6 M/0.1 M/0.1 M/35 ml/65 ml) solution and Ni was electrodeposited cathodically by repeating pulses at −0.85 V at 2 sec. intervals in a three-electrode system. An FTO substrate was used as the working electrode, a platinum plate as the counter electrode and Ag/AgCl (in saturated KCl aqueous solution) as a reference electrode. The PS nanospheres were then removed by immersing the Ni/PS-covered substrate into a tetrahydrofuran (THF, ≥99%, Daejung Chemical and Metals Co. Ltd.) solution with stirring for 6 h.

### Electrodeposition of MnO_2_ on 3D Ni nanostructures

The electrodeposition of MnO_2_ onto the 3D Ni inverse-opal nanostructures was performed in the three electrode system described earlier. A cyclic voltammetric technique was used by repeating potential cycles of between 0.4 V and 1.3 V at a scan rate of 30 mV s^−1^ in 2.5 mM manganese sulfate monohydrate (MnSO_4_·H_2_O, ≥99%, Sigma-Aldrich) and 2.5 mM sodium acetate (CH_3_COONa, ≥ 99%, Sigma-Aldrich) in an ethanol/H_2_O (35 ml/65 ml) solution. For comparison, MnO_2_ layers on a planar Ni film-coated FTO substrate were also fabricated using the same electrodeposition procedure. After deposition, the electrodes were washed with ethanol and distilled water, followed by annealing at 60 °C for 2 h. The weight of the electrodeposited MnO_2_ was determined by a quartz crystal microbalance (QCM, Stanford Research Systems QCM200).

### Characterization

The crystalline structures of the electrodeposited Ni and MnO_2_ were observed by using x-ray diffraction (XRD, Philips PW1827), as shown in Figure S1. The fabricated nanostructures and surface morphologies of the Ni current collectors and MnO_2_ electrodes were characterized by FE-SEM (JSM-7410F, JEOL Ltd.). The electrochemical properties were evaluated by cyclic voltammetry and galvanostatic charge-discharge technique in a 1.0 M aqueous Na_2_SO_4_ solution at room temperature using a cyclic voltammeter (ZIVE SP2, WonATech). The EIS was also measured over the frequency range from 10^−1^ to 10^5^ Hz at open-circuit potential with amplitude of 10 mV.

## Electronic supplementary material

Supplementary Information
