# Supplementary material for: Retarded saturation of the areal capacitance using 3D-aligned MnO2 thin film nanostructures as a supercapacitor electrode
Source: Sci Rep. 2017 Aug 15;7:8260. doi: 10.1038/s41598-017-09039-x (PMC5557763; doi:10.1038/s41598-017-09039-x)
Supplement: Supplementary file 1 — Supplementary Information [file 41598_2017_9039_MOESM1_ESM.pdf]

Supplementary Information

Retarded saturation of areal capacitance using 3D-aligned MnO_2 thin film nanostructures as a supercapacitor electrode

Green Kim, Ilhwan Ryu and Sanggyu Yim*

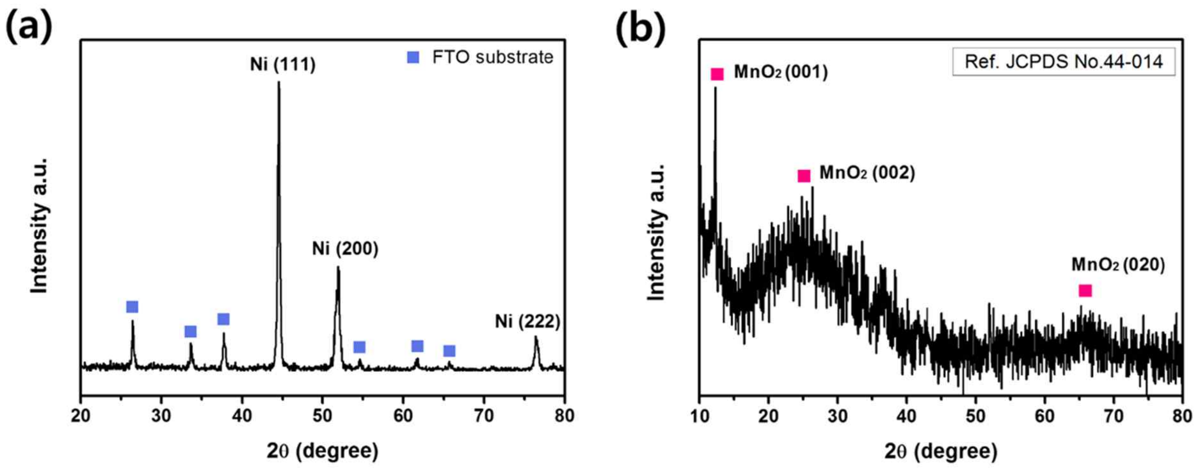

Figure S1**.** Powder X-ray diffraction patterns of electrodeposited (a) Ni and (b) MnO_2 thin films. The diffraction peaks indexed are consistent with standard data from JCPDS.

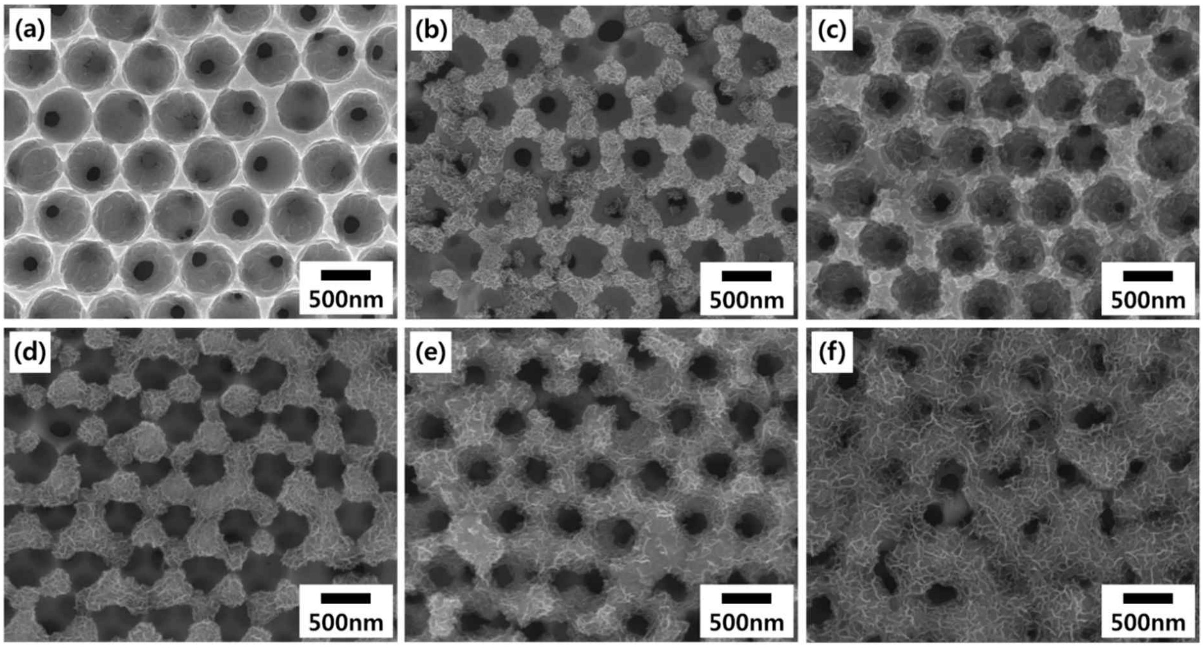

Figure S2. Surface FE-SEM images of MnO_2 thin films electrodeposited on 3Lyr inverse-opal Ni nanostructures. The number of electrodeposition cycles is (a) 5, (b) 10, (c) 15, (d) 20, (e) 25 and (f) 30.

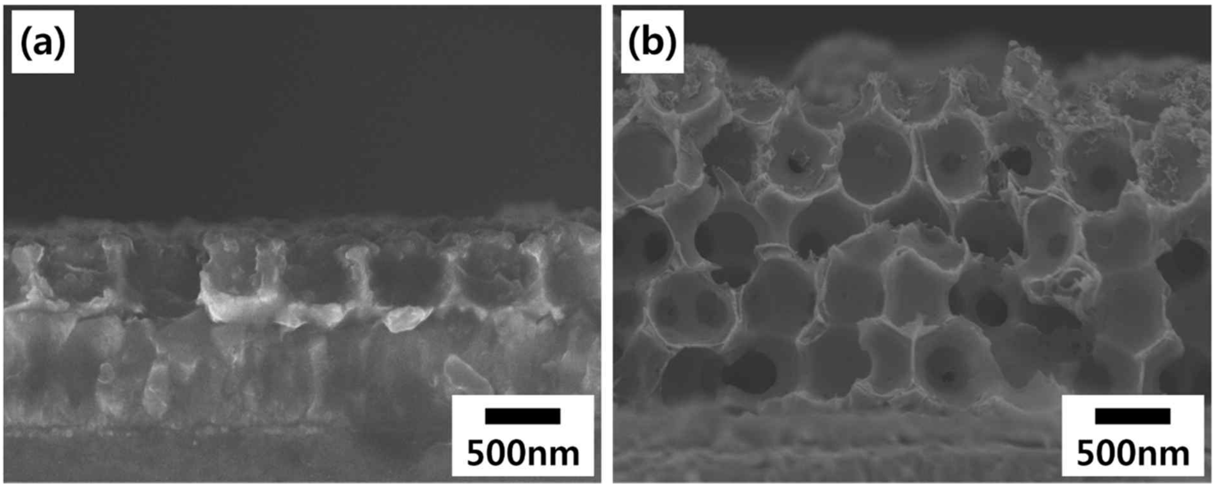

Figure S3. Cross-sectional FE-SEM images after 15 cycle electrodeposition of MnO_2 on the (a) 1Lyr- and (b) 5-Lyr Ni nanostructures.

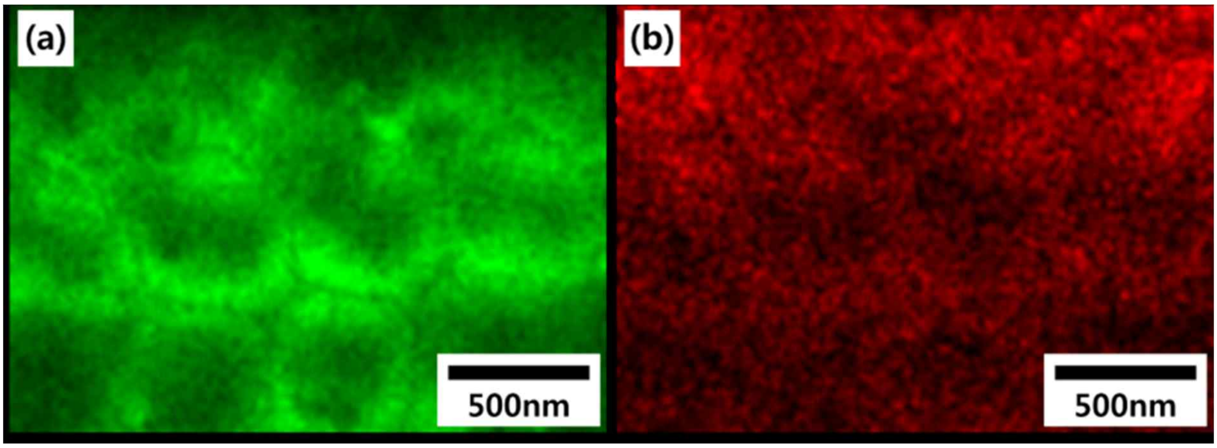

Figure S4. Cross-sectional SEM-mapping images for (a) Ni and (b) Mn atoms taken after 10 cycle electrodeposition of MnO_2 on the 3Lyr Ni nanostructures.

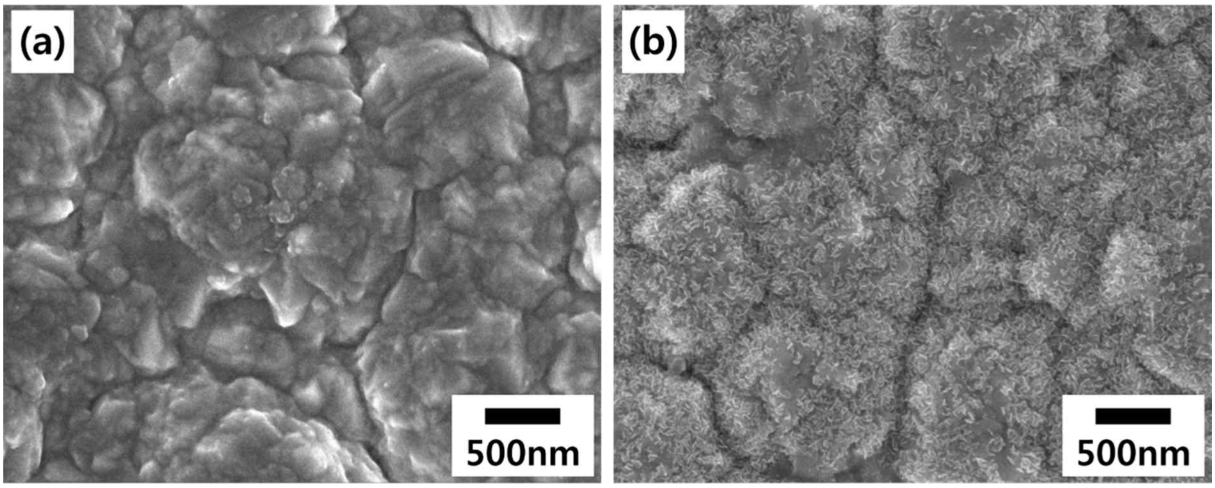

Figure S5. Surface FE-SEM images of the (a) planar Ni film and (b) MnO_2 layer electrodeposited on it.

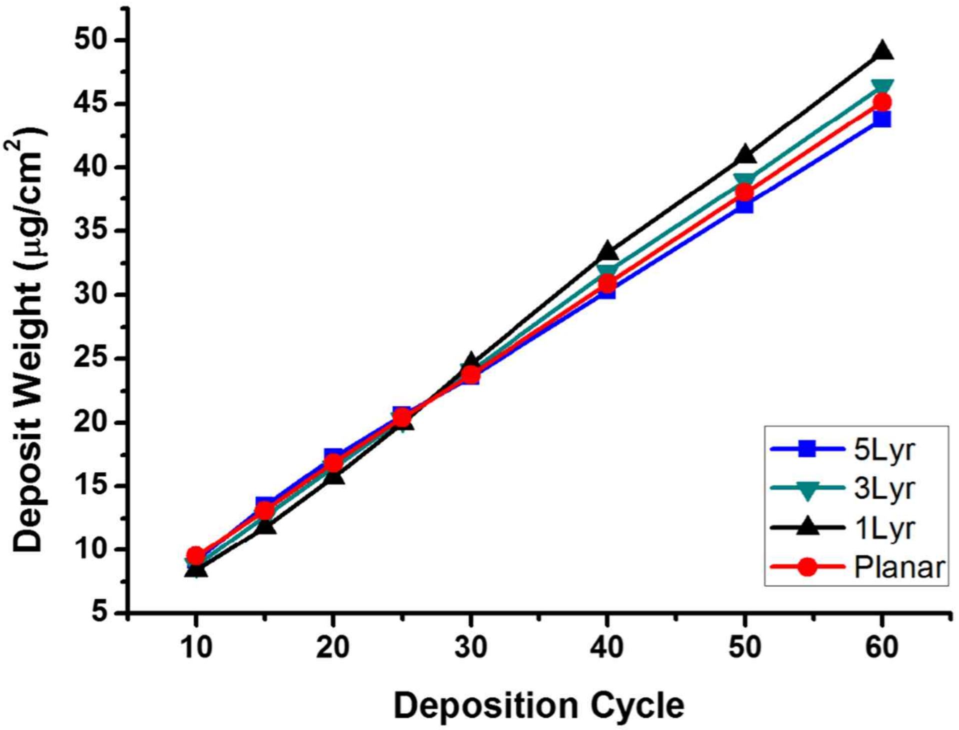

Figure S6. Plots of deposit weights of MnO_2 thin films electrodeposited on the planar and nanostructured Ni current collectors as a function of the number of electrodeposition cycles.

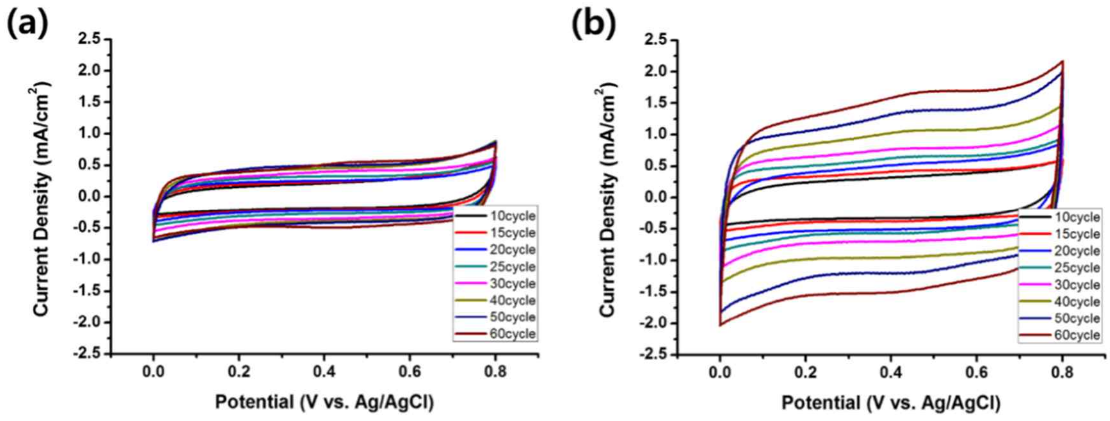

Figure S7. Cyclic voltammograms of MnO_2 electrodes deposited on the (a) planar and (b) 5Lyr nanostructured Ni surface. The scan rate was 100 mV s^{-1} for both samples.

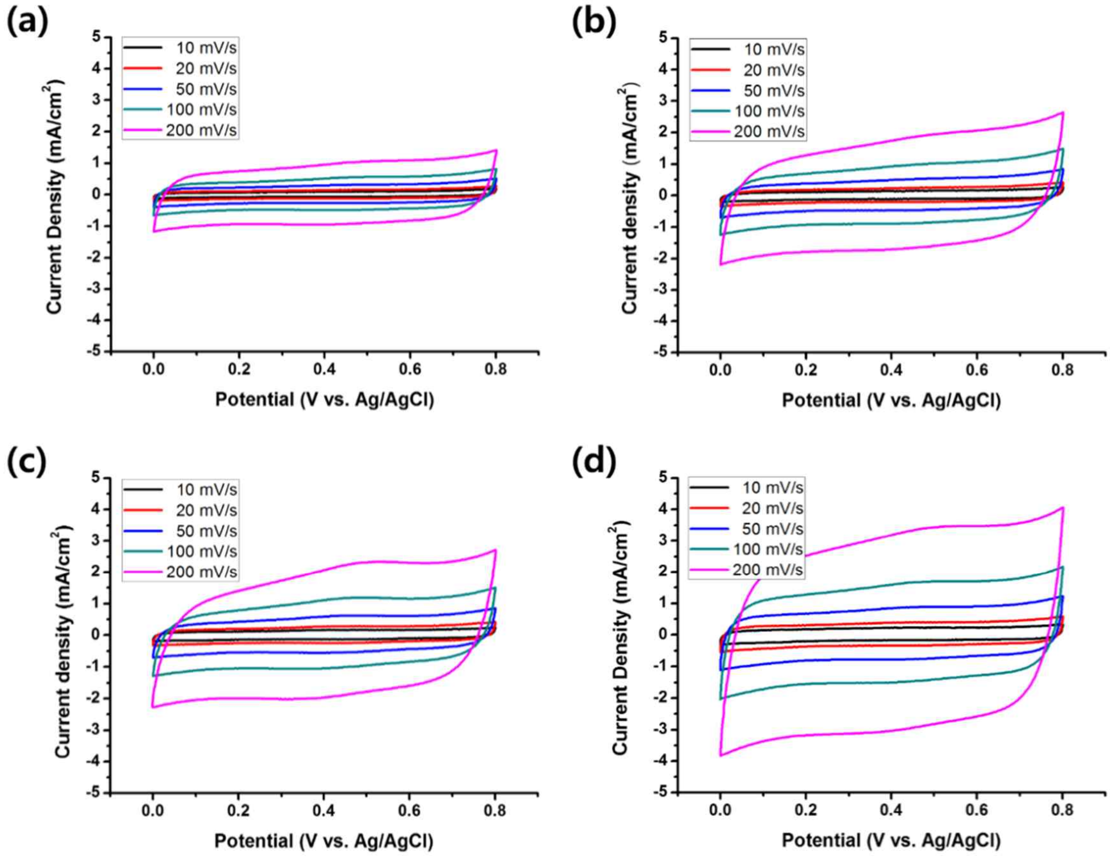

Figure S8. Cyclic voltammograms of the (a) planar, (b) 1Lyr-, (c) 3Lyr-, and (d) 5Lyr-nanostructured MnO_2 electrodes at various scan rates. The number of MnO_2 electrodeposition cycles was 60 for all samples.

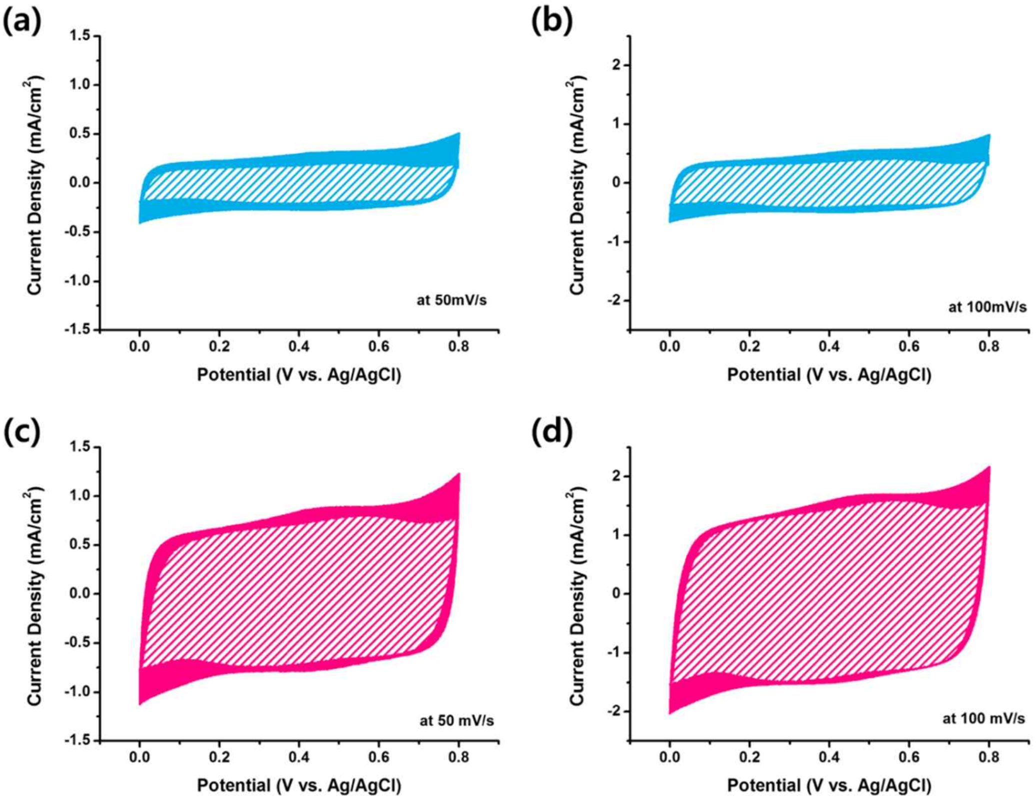

Figure S9. Deconvolution of the total capacitance into the surface capacitive (shaded part) and insertion (solid part) elements for the planar MnO_2 electrode at scan rates of (a) 50 mV s^{-1} and (b) 100 mV s^{-1}, and for the 5Lyr nanostructured MnO_2 electrode at scan rates of (c) 50 mV s^{-1} and (d) 100 mV s^{-1}.
